# Supplementary material for: Partitioning the impact of environment and spatial structure on alpha and beta components of taxonomic, functional, and phylogenetic diversity in European ants
Source: PeerJ. 2015 Sep 29;3:e1241. doi: 10.7717/peerj.1241 (PMC4592154; doi:10.7717/peerj.1241)
Supplement: Appendix S1 [file peerj-03-1241-s001.doc]

(many of the references have been obtained from AntWeb. Available from http://www.antweb.org. Accessed 10 January 2014)

Agosti, D. (1990) Review and reclassification of *Cataglyphis* (Hymenoptera, Formicidae). *Journal of Natural History*, **24**, 1457-1505.

Beibl, J., Buschinger, A., Foitzik, S. & Heinze, J. (2007) Phylogeny and phylogeography of the Mediterranean species of the parasitic ant genus *Chalepoxenus* and its *Temnothorax* hosts. *Insectes Sociaux*, **54**, 189-199.

Bernard, F. (1950) ("1946"). Notes sur les fourmis de France. II. Peuplement des montagnes méridionales. *Annales de la Société Entomologique de France*, **115**, 1-36.

Bernard, F. (1968) Les Fourmis (Hymenoptera Formicidae) d'Europe Occidentales et Septentrionale. Masson et Cie éditeurs, Paris. 411pp.

Brunner, E., Kroiss, J., Trindl, A. & Heinze, J. (2011) Queen pheromones in *Temnothorax* ants: control or honest signal? *BMC Evolutionary Biology*, **11**, 55. http://www.biomedcentral.com/1471-2148/11/55.

Buschinger, A. (1966) *Leptothorax* (*Mychothorax*) *muscorum* Nylander und Leptothorax (*M.)* *gredleri* Mayr zwei gute Arten. *Insectes Sociaux*, **13**, 165-172.

Cagniant H, Espadaler X (1997). Les *Leptothorax*, *Epimyrma* et *Chalepoxenus* du Maroc (Hymenoptera: Formicidae). Clé et catalogue des espèces. *Annales de la Société Entomologique de France (NS)*, **33**, 259-284.

Espadaler, X. (1996) Diagnosis preliminar de siete especies nuevas de hormigas de la Península Ibérica (Hymenoptera: Formicidae). *Zapateri*, **6**, 151-153.

Espadaler, X. (1997) Redescription of *Leptothorax schaufussi* (Forel, 1879) (Hymenoptera: Formicidae). *Orsis*, **12**, 101-107.

Goropashnaya, A.V., Fedorov, V.B., Seifert, B. & Pamilo, P. (2012) Phylogenetic relationships of palaearctic *Formica* species (Hymenoptera, Formicidae) based on mitochondrial cytochrome b sequences. *PLoS ONE*, **7**, e41697. doi:10.1371/journal.pone.0041697

Jansen, G., Savolainen, R. & Vepsäläinen, K. (2009) DNA barcoding as a heuristic tool for classifying undescribed Nearctic *Myrmica* ants (Hymenoptera: Formicidae). *Zoologica Scripta*, **38**, 527-536.

Machac, A., Janda, M., Dunn, R.R. & Sanders, N.J. (2011) Elevational gradients in phylogenetic structure of ant communities reveal the interplay of biotic and abiotic constraints on species density. *Ecography*, **34**, 364-371.

Maruyama, M., Steine, F.M., Stauffer, C., Akino, T., Crozier, R.H., Schlick-Steiner, B.C. (2008) A DNA and morphology based phylogenetic framework of the ant genus *Lasius* with hypotheses for the evolution of social parasitism and fungiculture. *BMC Evolutionary Biology*, **8**, 237. doi:10.1186/1471-2148-8-237

Moreau, C.S., Bell, C.D., Vila, R., Archibald, S.B. & Pierce, N.E. (2006) Phylogeny of the ants: diversification in the age of angiosperms. *Science*, **312**, 101-104.

Oettler, J., Suefuji, M. & Heinze, J. (2010) The evolution of alternative reproductive tactics in *Cardiocondyla* male ants. *Evolution*, **64**, 3310-3317.

Reyes, J.L., Espadaler, X. & Rodríguez, A. (1987) Descripción de *Goniomma baeticum* nov. sp. (Hym., Formicidae). *Eos*, **63**, 269-276.

Rigato, F. (2011) Contributions to the taxonomy of West European and North African *Stenamma* of the *westwoodii* species-group. (Hymenoptera Formicidae). *Memorie della Società Italiana di Scienze Naturali e del Museo Civico di Storia Naturale di Milano*, **37**, 1-56.

Sanetra, M., Güsten, R. & Schulz, A. (1999) On the taxonomy and distribution of the Italian *Tetramorium* species and their social parasites (Hymenoptera Formicidae). *Memorie della Società Entomologica Italiana*, **77**, 317-357.

Schlick-Steiner BC, Steiner FM, Moder K, Seifert B, Sanetra M, Dyreson E, Stauffer C, Christian E (2006) A multidisciplinary approach reveals cryptic diversity in western Palearctic *Tetramorium* ants (Hymenoptera: Formicidae). *Molecular Phylogenetics and Evolution*, **40**, 259-273.

Seifert, B. (1992) A taxonomic revision of the Palaearctic members of ant subgenus *Lasius* s.str. (Hymenoptera: Formicidae). *Abh. Ber. Naturkundemus. Görlitz*, **66**, 1-67.

Seifert, B., Schlick-Steiner, B.C. & Steiner, F.M. (2009) *Myrmica constricta* Karavajev, 1934 - a cryptic species of *Myrmica hellenica* Finzi, 1926 (Hymenoptera: Formicidae). *Soil Organisms*, **81**, 53-76.

Seifert, B. & Schultz, R. (2009) A taxonomic revision of the *Formica rufibarbis* Fabricius, 1793 group (Hymenoptera: Formicidae). *Myrmecological News*, **12**, 255-272.

Tinaut, A. (1991) [1990]. Situación taxonómica del género *Cataglyphis* Förster, 1850 en la Península Ibérica. III. El grupo de *C. velox* Santschi, 1929 y descripción de *Cataglyphis humeya* sp. n. (Hymenoptera, Formicidae). *EOS*, **66**, 215-227.

Tinaut, A. (1993) *Cataglyphis floricola* nov. sp. new species for the genus *Cataglyphi*s Förster, 1850 (Hymenoptera, Formicidae) in the Iberian Peninsula. *Mitteilungen der Schweizerischen entomologischen Gesellschaf*, **66**, 123-134.

Tinaut, A., Ruano, F., Hidalgo, J. & Ballesta, M. (1994) Mirmecocenosis del sistema de dunas del Paraje Natural Punta Entinas-El Sabinar (Almería) (Hymenoptera Formicidae) Aspectos taxonómicos functionales y biogeográficos. *Graellsia*, **50**, 71-84.

Ward, P.S. (2007) Phylogeny, classification, and species-level taxonomy of ants (Hymenoptera: Formicidae). *Zootaxa*, **1668**, 549-563.
