# Supplementary figures and images for: Partitioning the impact of environment and spatial structure on alpha and beta components of taxonomic, functional, and phylogenetic diversity in European ants
Source: PeerJ. 2015 Sep 29;3:e1241. doi: 10.7717/peerj.1241 (PMC4592154; doi:10.7717/peerj.1241)

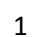

Supplement: Fig. S1 — Phylogenetic tree for the 155 European ant species examined in this study. [file peerj-03-1241-s002.pdf]

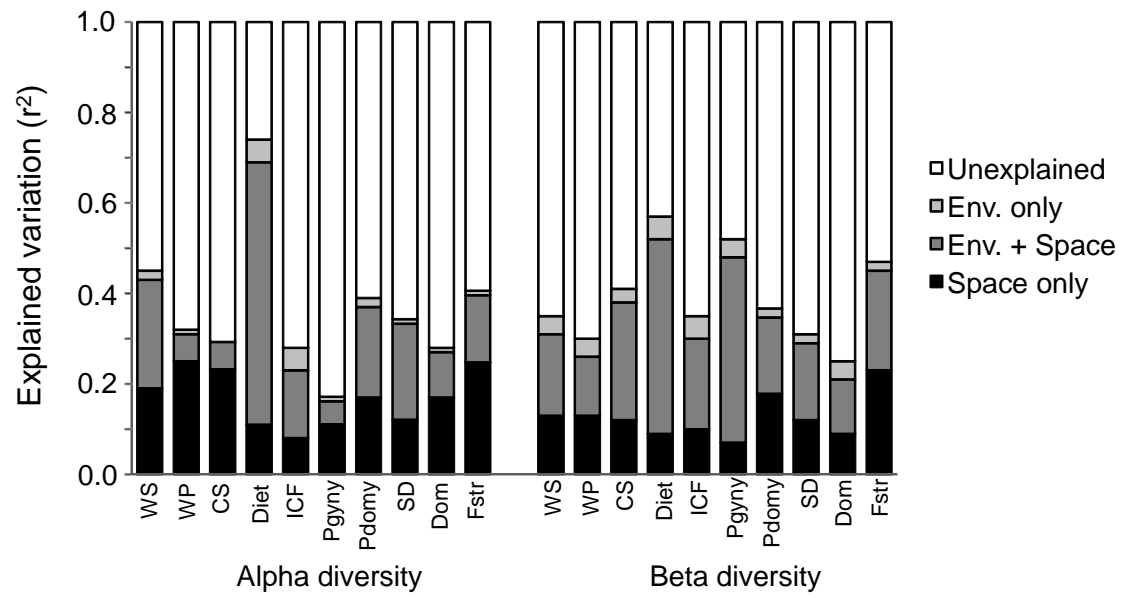

Supplement: Fig. S2 — The figure depicts the adjusted unique contribution (r2) of spatial components (black), interactions of spatial and environmental components (dark grey), environmental components; Env (light grey), and the variation left unexplained (white), computed by RDA. Abbreviations, according to Appendix S2 Table 1. Tests of significance (RDA) for unique environmental and spatial fractions shown that in all cases they were significant (p < 0.05), excepting for the alpha components of functional diversity in colony size and foraging strategy. [file peerj-03-1241-s003.pdf]
