# Supplementary material for: Partitioning the impact of environment and spatial structure on alpha and beta components of taxonomic, functional, and phylogenetic diversity in European ants
Source: PeerJ. 2015 Sep 29;3:e1241. doi: 10.7717/peerj.1241 (PMC4592154; doi:10.7717/peerj.1241)
Supplement: Table S1 — Abbreviations: WS, worker size; WP, worker polymorphism; Diur, diurnality; Dom, behavioral dominance; pS, proportion of seeds in diet; pI, proportion of insects in diet; pLF, proportion of liquid food in diet; FSI, individual foraging strategy; FSG, group foraging strategy; FSC, collective foraging strategy; CS, colony size (ln-transformed); nQ, number of queens; nN, number of nests; CFT, colony foundation type. The states of the different functional traits are shown in Table S2. [file peerj-03-1241-s004.pdf]

| SPECIES | WS | WP | Diur | Dom | pS | pI | pLF | FSI | FSG | FSC | CS | nQ | nN | CFT |
|---|---|---|---|---|---|---|---|---|---|---|---|---|---|---|
| **Formicinae** | | | | | | | | | | | | | | |
| *Camponotus aethiops* | 7.50 | 0.67 | 0 | 1 | 0 | 0.25 | 0.75 | 0 | 1 | 0 | 7.47 | 0 | 0 | 1 |
| *Camponotus amaurus* | 6.10 | 0.69 | 1 | 0 | 0 | 0 | 1 | 0 | 1 | 0 | 5.99 | 0 | 1 | 1 |
| *Camponotus cruentatus* | 10.00 | 0.80 | 0 | 1 | 0 | 0.25 | 0.75 | 0 | 1 | 0 | 8.52 | 0 | 0 | 1 |
| *Camponotus gestroi* | 6.10 | 0.59 | 0 | 0 | 0 | 0 | 1 | 0 | 1 | 0 | 6.21 | 0 | 0 | 1 |
| *Camponotus fallax* | 6.90 | 0.41 | 0 | 0 | 0 | 0 | 1 | 0 | 1 | 0 | 6.21 | 0 | 0 | 1 |
| *Camponotus figaro* | 3.90 | 0.44 | 1 | 0 | 0 | 0 | 1 | 0 | 1 | 0 | 6.21 | 0 | 0 | 1 |
| *Camponotus foreli* | 6.10 | 0.69 | 1 | 0 | 0 | 0 | 1 | 0 | 1 | 0 | 5.99 | 0 | 1 | 1 |
| *Camponotus herculeanus* | 10.80 | 0.69 | 0 | 1 | 0 | 0.25 | 0.75 | 0 | 1 | 0 | 8.52 | 0.5 | 0 | 1 |
| *Camponotus lateralis* | 5.00 | 0.40 | 1 | 0 | 0 | 0 | 1 | 0 | 1 | 0 | 6.91 | 0 | 0 | 1 |
| *Camponotus ligniperdus* | 9.00 | 0.67 | 0 | 1 | 0 | 0.25 | 0.75 | 0 | 1 | 0 | 7.82 | 0.5 | 0 | 1 |
| *Camponotus micans* | 8.50 | 1.29 | 0 | | 0 | 0 | 1 | 0 | 1 | 0 | 6.91 | 0 | 0 | 1 |
| *Camponotus piceus* | 3.90 | 0.44 | 1 | 0 | 0 | 0 | 1 | 0 | 1 | 0 | 6.21 | 0 | 0 | 1 |
| *Camponotus pilicornis* | 8.50 | 0.82 | 0 | 1 | 0 | 0 | 1 | 0 | 1 | 0 | 6.91 | 0 | 0 | 1 |
| *Camponotus sylvaticus* | 8.00 | 0.75 | 0 | 1 | 0 | 0 | 1 | 0 | 1 | 0 | 6.91 | 0 | 0 | 1 |
| *Camponotus truncatus* | 4.50 | 0.67 | 0 | 0 | 0 | 0 | 1 | 1 | 0 | 0 | 5.70 | 0 | 0 | 1 |
| *Camponotus vagus* | 9.40 | 0.68 | 0 | 1 | 0 | 0.25 | 0.75 | 0 | 1 | 0 | 7.82 | 1 | 0 | 1 |
| *Cataglyphis aenescens* | 5.55 | 0.88 | 1 | 0 | 0 | 1 | 0 | 1 | 0 | 0 | 6.91 | 0 | 0 | 1 |
| *Cataglyphis cursor* | 6.70 | 0.61 | 1 | 0 | 0 | 1 | 0 | 1 | 0 | 0 | 6.62 | 0 | 0 | 0 |
| *Cataglyphis floricola* | 6.00 | 0.22 | 1 | 0 | 0 | 1 | 0 | 1 | 0 | 0 | 5.52 | 0 | 0 | 0 |
| *Cataglyphis hispanica* | 8.50 | 0.93 | 1 | 0 | 0 | 1 | 0 | 1 | 0 | 0 | 6.62 | 0 | 0 | 0 |
| *Cataglyphis iberica* | 6.00 | 0.50 | 1 | 0 | 0 | 1 | 0 | 1 | 0 | 0 | 6.48 | 0 | 1 | 1 |
| *Cataglyphis rosenhaueri* | 6.00 | 0.50 | 1 | 0 | 0 | 1 | 0 | 1 | 0 | 0 | 6.40 | 0 | 1 | 1 |
| *Cataglyphis velox* | 8.30 | 0.90 | 1 | 0 | 0 | 1 | 0 | 1 | 0 | 0 | 6.62 | 0.5 | 0.5 | 0 |
| *Formica aquilonia* | 6.60 | 0.76 | 0 | 1 | 0 | 0.5 | 0.5 | 0 | 0 | 1 | 12.90 | 1 | 1 | 0 |
| *Formica cinerea* | 5.30 | 0.66 | 0 | 1 | 0 | 0.5 | 0.5 | 0 | 0 | 1 | 8.52 | 0.5 | 1 | 0 |
| *Formica clara* | 6.50 | 0.46 | 1 | 1 | 0 | 0.5 | 0.5 | 0 | 1 | 0 | 8.52 | 0 | 0 | 1 |
| *Formica cunicularia* | 5.30 | 0.47 | 1 | 0 | 0 | 0.5 | 0.5 | 0 | 1 | 0 | 7.24 | 0 | 0.5 | 1 |
| *Formica decipiens* | 5.60 | 0.41 | 1 | 0 | 0 | 0.5 | 0.5 | 0 | 1 | 0 | 7.13 | 0 | 0 | 1 |
| *Formica fusca* | 5.00 | 0.60 | 1 | 0 | 0 | 0.5 | 0.5 | 0 | 1 | 0 | 9.10 | 1 | 1 | 1 |
| *Formica gagates* | 5.00 | 0.60 | 1 | 0 | 0 | 0.5 | 0.5 | 0 | 1 | 0 | 6.21 | 0 | 0 | 1 |
| *Formica gerardi* | 5.50 | 0.36 | 1 | 0 | 0 | 0.5 | 0.5 | 0 | 1 | 0 | 7.13 | 0 | 0 | 1 |
| *Formica lemani* | 5.50 | 0.38 | 1 | 0 | 0 | 0.5 | 0.5 | 0 | 1 | 0 | 7.47 | 0 | 0 | 1 |
| *Formica lugubris* | 6.50 | 0.77 | 0 | 1 | 0 | 0.5 | 0.5 | 0 | 0 | 1 | 10.60 | 1 | 1 | 0.5 |
| *Formica lusatica* | 6.50 | 0.46 | 1 | 1 | 0 | 0.5 | 0.5 | 0 | 1 | 0 | 8.52 | 0 | 0 | 1 |
| *Formica nigricans* | 6.50 | 0.77 | 0 | 1 | 0 | 0.5 | 0.5 | 0 | 0 | 1 | 11.00 | 1 | 1 | 0 |
| *Formica polyctena* | 6.50 | 0.77 | 0 | 1 | 0 | 0.5 | 0.5 | 0 | 0 | 1 | 13.02 | 1 | 1 | 0 |
| *Formica pratensis* | 6.50 | 0.77 | 0 | 1 | 0 | 0.5 | 0.5 | 0 | 0 | 1 | 11.00 | 1 | 1 | 0 |
| *Formica rufa* | 6.50 | 0.77 | 0 | 1 | 0 | 0.5 | 0.5 | 0 | 0 | 1 | 9.21 | 1 | 1 | 0 |
| *Formica rufibarbis* | 6.00 | 0.50 | 1 | 0 | 0 | 0.5 | 0.5 | 0 | 1 | 0 | 6.91 | 0 | 0 | 1 |
| *Formica sanguinea* | 7.50 | 0.40 | 1 | 1 | 0 | 0.5 | 0.5 | 0 | 0 | 1 | 9.21 | 0.5 | 0 | 0.5 |
| *Formica subrufa* | 4.90 | 0.29 | 1 | 0 | 0 | 0.75 | 0.25 | 0 | 1 | 0 | 6.55 | 0 | 0 | 1 |
| *Formica transkaucasica* | 3.90 | 0.62 | 0 | 0 | 0 | 0.5 | 0.5 | 0 | 1 | 0 | 7.60 | 0 | 1 | 0 |
| *Formica truncorum* | 6.30 | 0.87 | 0 | 1 | 0 | 0.5 | 0.5 | 0 | 0 | 1 | 9.21 | 1 | 1 | 0 |
| *Formica uralensis* | 6.30 | 0.40 | 0 | 0 | 0 | 0.5 | 0.5 | 0 | 0 | 1 | | 1 | 1 | 0 |
| *Lasius alienus* | 2.80 | 0.50 | 0 | 1 | 0 | 0.25 | 0.75 | 0 | 0 | 1 | 9.47 | 0 | 0 | 1 |
| *Lasius balcanicus* | 5.50 | 0.36 | 0 | | 0 | 0.25 | 0.75 | 0 | 0 | 1 | 9.90 | 0 | 0 | 1 |
| *Lasius brunneus* | 2.90 | 0.62 | 0 | 0 | 0 | 0.25 | 0.75 | 0 | 0 | 1 | 9.21 | 0 | 0 | 1 |
| *Lasius cinereus* | 3.30 | 0.45 | 0 | 1 | 0 | 0 | 1 | 0 | 0 | 1 | 9.21 | 0 | 0 | 1 |

| | | | | | | | | | | | | | | |
|---|---|---|---|---|---|---|---|---|---|---|---|---|---|---|
| *Lasius emarginatus* | 3.20 | 0.47 | 0 | 1 | 0 | 0.25 | 0.75 | 0 | 0 | 1 | 9.21 | 0 | 0 | 1 |
| *Lasius flavus* | 2.90 | 0.79 | 0 | 0 | 0 | 0.25 | 0.75 | 0 | 0 | 1 | 9.21 | 0.5 | 0 | 1 |
| *Lasius fuliginosus* | 4.00 | 0.50 | 0 | 1 | 0 | 0.25 | 0.75 | 0 | 0 | 1 | 14.73 | 0 | 0 | 0 |
| *Lasius grandis* | 3.50 | 0.57 | 0 | 1 | 0 | 0.25 | 0.75 | 0 | 0 | 1 | 9.21 | 0 | 0 | 1 |
| *Lasius lasioides* | 2.80 | 0.50 | 0 | 1 | 0 | 0.25 | 0.75 | 0 | 0 | 1 | 9.47 | 0 | 0 | 1 |
| *Lasius myops* | 2.90 | 0.79 | 0 | 0 | 0 | 0 | 1 | 0 | 0 | 1 | 8.52 | 0 | 0 | 1 |
| *Lasius niger* | 3.00 | 0.67 | 0 | 1 | 0 | 0.25 | 0.75 | 0 | 0 | 1 | 9.21 | 0 | 0 | 1 |
| *Lasius paralienus* | 4.50 | 0.44 | 0 | 1 | 0 | 0.25 | 0.75 | 0 | 0 | 1 | 9.21 | 0 | 0 | 1 |
| *Lasius psammophilus* | 3.40 | 0.59 | 0 | 0 | 0 | 0.25 | 0.75 | 0 | 0 | 1 | 10.43 | 0 | 1 | 1 |
| *Plagiolepis pygmaea* | 1.60 | 0.50 | 0 | 0 | 0 | 0 | 1 | 0 | 1 | 0 | 6.68 | 1 | 0.5 | 0 |
| *Plagiolepis schmitzii* | 2.10 | 0.48 | 1 | 0 | 0 | 0 | 1 | 0 | 1 | 0 | 6.68 | 1 | 0.5 | 0 |
| *Proformica ferreri* | 4.50 | 0.73 | 1 | 0 | 0 | 1 | 0 | 1 | 0 | 0 | 6.48 | 0 | 0 | |
| *Proformica nasuta* | 5.00 | 1.00 | 1 | 0 | 0 | 1 | 0 | 1 | 0 | 0 | 6.48 | 0 | 0 | |
| **Dolichoderinae** | | | | | | | | | | | | | | |
| *Dolichoderus quadripunctatus* | 3.50 | 0.29 | 0 | 0 | 0 | 0.5 | 0.5 | 0 | 0 | 1 | 6.11 | 0 | 1 | 0 |
| *Linepithema humile* | 2.30 | 0.22 | 0 | 1 | 0 | 0.25 | 0.75 | 0 | 0 | 1 | 11.92 | 1 | 1 | 0 |
| *Liometopum microcephalum* | 5.00 | 0.80 | 0 | 1 | 0 | 0.75 | 0.25 | 0 | 0 | 1 | 8.52 | 0 | 0 | 1 |
| *Tapinoma erraticum* | 2.80 | 0.54 | 0 | 1 | 0 | 0 | 1 | 0 | 0 | 1 | 8.16 | 1 | 1 | 0 |
| *Tapinoma nigerrimum* | 4.00 | 0.58 | 0 | 1 | 0 | 0 | 1 | 0 | 0 | 1 | 8.85 | 1 | 1 | 0 |
| *Tapinoma simrothi* | 3.20 | 0.44 | 0 | 1 | 0 | 0 | 1 | 0 | 0 | 1 | 8.85 | 1 | 1 | 0 |
| **Myrmicinae** | | | | | | | | | | | | | | |
| *Aphaenogaster dulcineae* | 6.00 | 0.33 | 0 | 0 | 0.5 | 0.5 | 0 | 0 | 1 | 0 | 6.48 | 0 | 0 | |
| *Aphaenogaster gibbosa* | 4.90 | 0.41 | 1 | 0 | 0.5 | 0.5 | 0 | 0 | 1 | 0 | 6.48 | 0 | 0 | 1 |
| *Aphaenogaster iberica* | 6.40 | 0.19 | 1 | 0 | 0.5 | 0.5 | 0 | 0 | 1 | 0 | 6.25 | 0 | 0 | 0 |
| *Aphaenogaster cardenai* | 6.70 | 0.10 | 0 | 0 | 0.5 | 0.5 | 0 | 0 | 1 | 0 | 8.01 | 0 | 0 | 1 |
| *Aphaenogaster senilis* | 7.00 | 0.14 | 1 | 0 | 0.5 | 0.5 | 0 | 0 | 1 | 0 | 6.48 | 0 | 0 | 0 |
| *Aphaenogaster subterranea* | 3.80 | 0.45 | 0 | 0 | 0.5 | 0.5 | 0 | 0 | 1 | 0 | 7.60 | 0 | 0 | 1 |
| *Cardiocondyla batesii* | 2.10 | 0.24 | 0 | 0 | 0 | 0.75 | 0.25 | 0 | 1 | 0 | 4.79 | 0 | 0 | 1 |
| *Cardiocondyla mauritanica* | 2.00 | 0.20 | 0 | 0 | 0 | 0.75 | 0.25 | 0 | 1 | 0 | 5.01 | 1 | 0 | 0 |
| *Crematogaster auberti* | 3.40 | 0.35 | 0 | 1 | 0 | 0 | 1 | 0 | 1 | 0 | 6.48 | 0 | 0 | 0 |
| *Crematogaster scutellaris* | 4.05 | 0.57 | 0 | 1 | 0 | 0.5 | 0.5 | 0 | 0 | 1 | 6.62 | 0.5 | 0.5 | 1 |
| *Crematogaster sordidula* | 2.45 | 0.37 | 0 | 1 | 0 | 0 | 1 | 0 | 1 | 0 | 6.62 | 0 | 0 | 1 |
| *Goniomma baeticum* | 4.10 | 0.24 | 0 | 0 | 1 | 0 | 0 | 1 | 0 | 0 | 5.86 | 0 | 0 | 1 |
| *Goniomma blanci* | 3.50 | 0.29 | 0 | 0 | 1 | 0 | 0 | 1 | 0 | 0 | 5.86 | 0 | 0 | 1 |
| *Goniomma collingwoodi* | 3.65 | 0.08 | 0 | 0 | 1 | 0 | 0 | 1 | 0 | 0 | 5.86 | 0 | 0 | 1 |
| *Goniomma hispanicum* | 3.90 | 0.21 | 0 | 0 | 1 | 0 | 0 | 1 | 0 | 0 | 5.86 | 0 | 0 | 1 |
| *Goniomma kugleri* | 3.10 | 0.19 | 0 | 0 | 1 | 0 | 0 | 1 | 0 | 0 | 5.89 | 0 | 0 | 1 |
| *Goniomma thoracicum* | 3.90 | 0.21 | 0 | 0 | 1 | 0 | 0 | 1 | 0 | 0 | 5.86 | 0 | 0 | 1 |
| *Leptothorax acervorum* | 3.60 | 0.17 | 0 | 0 | 0 | 0.5 | 0.5 | 0 | 1 | 0 | 7.60 | 1 | 0 | 0 |
| *Leptothorax gredleri* | 3.20 | 0.16 | 0 | 0 | 0 | 0.5 | 0.5 | 0 | 1 | 0 | 3.91 | 0 | 0 | 0.5 |
| *Leptothorax muscorum* | 2.90 | 0.55 | 0 | 0 | 0 | 0.5 | 0.5 | 0 | 1 | 0 | 5.70 | 1 | 0 | 0 |
| *Manica rubida* | 7.00 | 0.71 | 1 | 0 | 0 | 1 | 0 | 0 | 0 | 1 | 8.16 | 0.5 | 0 | 1 |
| *Messor barbarus* | 7.90 | 1.04 | 0 | 1 | 1 | 0 | 0 | 0 | 0 | 1 | 8.99 | 0 | 0 | 1 |
| *Messor celiae* | 7.90 | 1.04 | 0 | 1 | 1 | 0 | 0 | 0 | 0 | 1 | 8.99 | 0 | 0 | 1 |
| *Messor bouvieri* | 6.30 | 0.71 | 1 | 0 | 1 | 0 | 0 | 0 | 0 | 1 | 8.16 | 0 | 0 | 1 |
| *Messor maroccanus* | 6.30 | 0.71 | 1 | 0 | 1 | 0 | 0 | 0 | 0 | 1 | 8.16 | 0 | 0 | 1 |
| *Messor capitatus* | 8.40 | 1.07 | 0 | 1 | 1 | 0 | 0 | 0 | 1 | 0 | 8.29 | 0 | 0 | 1 |
| *Messor hispanicus* | 7.90 | 1.04 | 0 | 1 | 1 | 0 | 0 | 0 | 0 | 1 | 8.99 | 0 | 0 | 1 |
| *Messor lusitanicus* | 6.50 | 0.54 | 1 | 0 | 1 | 0 | 0 | 0 | 0 | 1 | 8.16 | 0 | 0 | 1 |
| *Messor structor* | 6.80 | 0.81 | 0 | 1 | 1 | 0 | 0 | 0 | 0 | 1 | 8.29 | 1 | 0 | 0 |
| *Monomorium salomonis* | 3.05 | 0.36 | 0 | 1 | 0.25 | 0.75 | 0 | 0 | 0 | 1 | 8.01 | 1 | 1 | 0 |
| *Myrmecina graminicola* | 3.05 | 0.36 | 0 | 0 | 0 | 0.75 | 0.25 | 1 | 0 | 0 | 4.61 | 0.5 | 0 | 0 |

| | | | | | | | | | | | | | | |
|---|---|---|---|---|---|---|---|---|---|---|---|---|---|---|
| *Myrmica aloba* | 4.50 | 0.22 | 0 | 0 | 0 | 0.5 | 0.5 | 0 | 0 | 1 | 7.60 | 0 | 0 | 0.5 |
| *Myrmica hellenica* | | | 0 | 0 | 0 | 0.5 | 0.5 | 0 | 0 | 1 | 7.38 | 0.5 | 0 | 0.5 |
| *Myrmica lobulicornis* | 3.75 | 0.27 | 0 | 0 | 0 | 0.5 | 0.5 | 0 | 0 | 1 | 7.31 | 1 | 0 | 0.5 |
| *Myrmica lonae* | 4.40 | 0.14 | 0 | 0 | 0 | 0.5 | 0.5 | 0 | 0 | 1 | 8.01 | 1 | 0 | 0.5 |
| *Myrmica rubra* | 4.00 | 0.25 | 0 | | 0 | 0.5 | 0.5 | 0 | 0 | 1 | 8.01 | 1 | 1 | 0.5 |
| *Myrmica ruginodis* | 4.75 | 0.11 | 0 | 0 | 0 | 0.5 | 0.5 | 0 | 0 | 1 | 7.60 | 0.5 | 0 | 0.5 |
| *Myrmica rugulosa* | 4.00 | 0.25 | 0 | 0 | 0 | 0.5 | 0.5 | 0 | 0 | 1 | 7.60 | 1 | 1 | 0.5 |
| *Myrmica sabuleti* | 4.40 | 0.14 | 0 | 0 | 0 | 0.5 | 0.5 | 0 | 0 | 1 | 8.01 | 1 | 0 | 0.5 |
| *Myrmica scabrinodis* | 4.25 | 0.24 | 0 | 0 | 0 | 0.5 | 0.5 | 0 | 0 | 1 | 7.31 | 0.5 | 0 | 0.5 |
| *Myrmica schencki* | 4.15 | 0.51 | 0 | 0 | 0 | 0.5 | 0.5 | 0 | 0 | 1 | 6.91 | 0.5 | 0 | 0.5 |
| *Myrmica specioides* | 5.60 | 0.18 | 0 | 0 | 0 | 0.5 | 0.5 | 0 | 0 | 1 | 7.31 | 1 | 0 | 0.5 |
| *Myrmica spinosior* | 4.40 | 0.14 | 0 | 0 | 0 | 0.5 | 0.5 | 0 | 0 | 1 | 8.01 | 1 | 0 | 0.5 |
| *Myrmica sulcinodis* | 4.75 | 0.32 | 0 | 0 | 0 | 0.5 | 0.5 | 0 | 0 | 1 | 6.68 | 0 | 0.5 | 0.5 |
| *Myrmica wesmaeli* | 3.80 | 0.26 | 0 | 0 | 0 | 0.5 | 0.5 | 0 | 0 | 1 | 7.31 | 1 | 0 | 0.5 |
| *Oxyopomyrmex saulcyi* | 2.00 | 0.20 | 0 | 0 | 1 | 0 | 0 | 1 | 0 | 0 | 4.61 | 0 | 0 | 1 |
| *Pheidole pallidula* | 3.20 | 1.03 | 0 | 1 | 0.25 | 0.75 | 0 | 0 | 0 | 1 | 8.52 | 0.5 | 0 | 0.5 |
| *Stenamma orousetti* | 3.30 | 0.18 | 0 | 0 | 0 | 1 | 0 | 1 | 0 | 0 | 4.61 | 0 | 0 | 1 |
| *Stenamma westwoodi* | 3.30 | 0.18 | 0 | 0 | 0 | 1 | 0 | 1 | 0 | 0 | 4.61 | 0 | 0 | 1 |
| *Stenamma petiolatum* | 4.50 | 0.13 | 0 | 0 | 0 | 1 | 0 | 1 | 0 | 0 | 4.61 | 0 | 0 | 1 |
| *Temnothorax angustulus* | 2.80 | 0.21 | 0 | 0 | 0 | 0.5 | 0.5 | 0 | 1 | 0 | 3.91 | 0 | 0 | 1 |
| *Temnothorax caesari* | 2.20 | 0.18 | 0 | 0 | 0 | 0.5 | 0.5 | 0 | 1 | 0 | 4.61 | 0 | 0 | |
| *Temnothorax clypeatus* | 3.00 | 0.33 | 0 | 0 | 0 | 0.5 | 0.5 | 0 | 1 | 0 | 4.61 | 0 | 0 | 1 |
| *Temnothorax crassispinus* | 2.90 | 0.41 | 0 | 0 | 0 | 0.5 | 0.5 | 0 | 1 | 0 | 4.61 | 0 | 0 | 0.5 |
| *Temnothorax exilis* | 2.60 | 0.46 | 0 | 0 | 0 | 0.5 | 0.5 | 0 | 1 | 0 | 3.91 | 0 | 0 | 1 |
| *Temnothorax fuentei* | 4.15 | 0.07 | 0 | 0 | 0 | 0.5 | 0.5 | 0 | 1 | 0 | 3.91 | 0 | 0 | 1 |
| *Temnothorax gredosi* | 2.50 | 0.32 | 0 | 0 | 0 | 0.5 | 0.5 | 0 | 1 | 0 | 5.01 | 0 | 0 | 1 |
| *Temnothorax grouvellei* | 2.85 | 0.46 | 0 | 0 | 0 | 0.5 | 0.5 | 0 | 1 | 0 | 4.61 | 0 | 0 | 1 |
| *Temnothorax interruptus* | 2.30 | 0.52 | 0 | 0 | 0 | 0.5 | 0.5 | 0 | 1 | 0 | 5.01 | 1 | 0 | 0 |
| *Temnothorax kraussei* | 2.80 | 0.21 | 0 | 0 | 0 | 0.5 | 0.5 | 0 | 1 | 0 | 3.91 | 0 | 0 | 1 |
| *Temnothorax lichtensteini* | 2.60 | 0.08 | 0 | 0 | 0 | 0.5 | 0.5 | 0 | 1 | 0 | 5.30 | 0 | 0 | 1 |
| *Temnothorax luteus* | 2.40 | 0.25 | 0 | 0 | 0 | 0.5 | 0.5 | 0 | 1 | 0 | | 0 | 0 | 1 |
| *Temnothorax niger* | 2.65 | 0.34 | 0 | 0 | 0 | 0.5 | 0.5 | 0 | 1 | 0 | 5.01 | 0 | 0 | 1 |
| *Temnothorax nigriceps* | 2.65 | 0.26 | 0 | 0 | 0 | 0.5 | 0.5 | 0 | 1 | 0 | 5.01 | 0 | 0 | 1 |
| *Temnothorax nylanderi* | 2.65 | 0.30 | 0 | 0 | 0 | 0.5 | 0.5 | 0 | 1 | 0 | 5.70 | 0 | 0 | 1 |
| *Temnothorax pardoi* | 2.50 | 0.08 | 0 | 0 | 0 | 0.5 | 0.5 | 0 | 1 | 0 | 5.01 | 0 | 0 | 1 |
| *Temnothorax parvulus* | 2.45 | 0.12 | 1 | 0 | 0 | 0.5 | 0.5 | 0 | 1 | 0 | 6.40 | 0 | 0 | 1 |
| *Temnothorax rabaudi* | 2.70 | 0.22 | 0 | 0 | 0 | 0.5 | 0.5 | 0 | 1 | 0 | 5.01 | 0 | 0 | 1 |
| *Temnothorax racovitzai* | 2.55 | 0.12 | 0 | 0 | 0 | 0.5 | 0.5 | 0 | 1 | 0 | 5.01 | 0 | 0 | 1 |
| *Temnothorax recedens* | 2.70 | 0.30 | 1 | 0 | 0 | 0.5 | 0.5 | 0 | 1 | 0 | 5.01 | 0 | 0 | 0 |
| *Temnothorax specularis* | 2.35 | 0.13 | 0 | 0 | 0 | 0.5 | 0.5 | 0 | 1 | 0 | 5.01 | 0 | 0 | 1 |
| *Temnothorax tristis* | 2.50 | 0.40 | 0 | 0 | 0 | 0.5 | 0.5 | 0 | 1 | 0 | 5.01 | 1 | 0 | 1 |
| *Temnothorax tuberum* | 2.85 | 0.39 | 0 | 0 | 0 | 0.5 | 0.5 | 0 | 1 | 0 | 5.30 | 0 | 0.5 | 1 |
| *Temnothorax thyndalei* | 2.65 | 0.19 | 0 | 0 | 0 | 0.5 | 0.5 | 0 | 1 | 0 | 5.01 | 0 | 0 | 1 |
| *Temnothorax unifasciatus* | 2.50 | 0.40 | 0 | 0 | 0 | 0.5 | 0.5 | 0 | 1 | 0 | 5.78 | 0 | 0 | 1 |
| *Tetramorium caespitum* | 2.90 | 0.41 | 0 | 1 | 0.25 | 0.75 | 0 | 0 | 0 | 1 | 9.21 | 0 | 0 | 1 |
| *Tetramorium impurum* | 2.90 | 0.41 | 0 | 1 | 0.25 | 0.75 | 0 | 0 | 0 | 1 | 9.21 | 0 | 0 | 1 |
| *Tetramorium forte* | 3.75 | 0.51 | 0 | 1 | 0.25 | 0.5 | 0.25 | 0 | 0 | 1 | 9.21 | 0 | 0 | 1 |
| *Tetramorium hispanicum* | 3.75 | 0.51 | 0 | 1 | 0.25 | 0.5 | 0.25 | 0 | 0 | 1 | 9.21 | 0 | 0 | 1 |
| *Tetramorium ruginode* | 3.75 | 0.51 | 0 | 1 | 0.25 | 0.5 | 0.25 | 0 | 0 | 1 | 9.21 | 0 | 0 | 1 |
| *Tetramorium semilaeve* | 3.60 | 0.33 | 0 | 1 | 0.25 | 0.75 | 0 | 0 | 0 | 1 | 9.21 | 1 | 0 | 1 |
| *Tetramorium punicum* | 3.60 | 0.33 | 0 | 1 | 0.25 | 0.75 | 0 | 0 | 0 | 1 | 9.21 | 1 | 0 | 1 |
| **Ponerinae** | | | | | | | | | | | | | | |

| | | | | | | | | | | | | | | |
|---|---|---|---|---|---|---|---|---|---|---|---|---|---|---|
| *Hypoponera eduardi* | 2.85 | 0.18 | 0 | 0 | 0 | 1 | 0 | 0 | 1 | 0 | 7.31 | 1 | 0 | 1 |
| *Hypoponera punctatissima* | 3.15 | 0.16 | 0 | 0 | 0 | 1 | 0 | 0 | 1 | 0 | 5.01 | 1 | 1 | 0.5 |
| *Ponera coarctata* | 2.95 | 0.31 | 0 | 0 | 0 | 1 | 0 | 0 | 1 | 0 | 4.61 | 1 | 0 | 0 |
| **Pseudomyrmicinae** | | | | | | | | | | | | | | |
| *Tetraponera allaborans* | | | | 0 | 0 | 0.75 | 0.25 | | | | 6.62 | 0 | 0 | 0.5 |
| **Leptanillinae** | | | | | | | | | | | | | | |
| *Leptanilla revelieri* | 1.15 | 0.09 | 0 | 0 | 0 | 1 | 0 | 0 | 1 | 0 | 5.70 | 0 | 0 | 0 |
