# Supplementary material for: Partitioning the impact of environment and spatial structure on alpha and beta components of taxonomic, functional, and phylogenetic diversity in European ants
Source: PeerJ. 2015 Sep 29;3:e1241. doi: 10.7717/peerj.1241 (PMC4592154; doi:10.7717/peerj.1241)
Supplement: Table S2 — ∗ Variable categories were coded using a fuzzy-coding technique. Scores ranged from ‘0’ (no consumption of a food resource) to ‘1’ (frequent consumption of a food resource); ∗∗ Individual: workers of these species are not able to communicate their nestmates the presence of a food source, they forage and collect food individually; Group: workers of these species are able to communicate and guide a low number of nestmates to a previously discovered food source; Collective: workers of these species follow “anonymous” chemical signals provided by other nestmates to exploit a food source, they can organize mass-recruitment or temporal or permanent trails to the food source. [file peerj-03-1241-s005.pdf]

**Trait**	**Data type**	**States**
Worker size	Continuous	Worker body size from the tip of mandibles to tip of the gaster (mm)
Worker polymorphism	Continuous	Mean worker size divided by the range of worker size
Diurnality	Binary	(0) Non-strictly diurnal<br>(1) Strictly diurnal
Behavioral dominance	Binary	(0) Subordinate<br>(1) Dominant
Diet: Seed-eating, Insect-eating, and Liquid-food eating	Fuzzy-coded (*)	0-1 (for each of the three categories)
Foraging strategy: Individual, Group, and Collective (**)	Binary	0-1 (for each of the three categories)
Colony size	Continuous	Mean number of workers per colony
Number of queens	Ordinal	(0) Monogyny; (0.5) Both monogyny and polygyny; (1) Polygyny
Number of nests	Ordinal	(0) Monodomy; (0.5) Both monodomy and polydomy; (1) Polydomy
Colony foundation type	Ordinal	(0) DCF; (0.5) Both DCF and ICF; (1) ICF
