# Supplementary material for: Partitioning the impact of environment and spatial structure on alpha and beta components of taxonomic, functional, and phylogenetic diversity in European ants
Source: PeerJ. 2015 Sep 29;3:e1241. doi: 10.7717/peerj.1241 (PMC4592154; doi:10.7717/peerj.1241)
Supplement: Table S3 — Abbreviations: WS, worker size; WP, worker polymorphism; CS, colony size; ICF, independent colony founding; Pgyny, polygyny; Pdomy, polydomy; SD, strictly diurnals; Dom, Dominants; FSTR, foraging strategy. [file peerj-03-1241-s006.pdf]

	Alpha diversity									
	**WS**	**WP**	**CS**	**Diet**	**ICF**	**Pgyny**	**Pdomy**	**SD**	**Dom**	**FSTR**
**Mean annual temperature**	(+) 0.14 ***	(+) 0.06 ***		(+) 0.53 ***		(-) 0.04 ***	(-) 0.15 ***	(+) 0.05 ***	(+) 0.07 ***	(+) 0.04 ***
**Temperature amplitude**	(+) 0.09 ***	(+) 0.01 *		(+) 0.10 ***	(+) 0.02 **		(-) 0.01 *	(+) 0.03 ***	(+) 0.01 *	(+) 0.04 ***
**Annual precipitation**			(-) 0.04 ***			(-) 0.03 **				(-) 0.08 ***
**Precipitation seasonality**			(-) 0.01 *		(+) 0.18 ***		(+) 0.06 ***	(+) 0.14 ***		
**Land-uses diversity index**			(+) 0.01 *							
**Anthropogenic disturbance index**	(-) 0.03 ***							(-) 0.01 *	(-) 0.03 **	
**Full model adjusted r^2**	0.26	0.07	0.06	0.63	0.20	0.07	0.22	0.23	0.11	0.16

Table S2. Continuation.

	Beta diversity									
	**WS**	**WP**	**CS**	**Diet**	**ICF**	**Pgyny**	**Pdomy**	**SD**	**Dom**	**FSTR**
**Mean annual temperature**	0.05 ***	0.04 ***	0.28 ***	0.42 ***	0.13 ***	0.43 ***	0.04 ***	0.02 ***	0.09 ***	0.23 ***
**Temperature amplitude**	0.14 ***	0.03 ***		0.06 ***	0.01 *		0.12 ***		0.07 ***	
**Annual precipitation**		0.08 ***								
**Precipitation seasonality**	0.01 *	0.01 **	0.01 **		0.11 ***	0.02 ***	0.01 *	0.17 ***		
**Land-uses diversity index**							0.02 **			
**Anthropogenic disturbance index**	0.02 **	0.01 **								0.01 *
**Full model adjusted r^2**	0.22	0.17	0.29	0.48	0.25	0.45	0.19	0.19	0.016	0.24
