# Supplementary material for: Partitioning the impact of environment and spatial structure on alpha and beta components of taxonomic, functional, and phylogenetic diversity in European ants
Source: PeerJ. 2015 Sep 29;3:e1241. doi: 10.7717/peerj.1241 (PMC4592154; doi:10.7717/peerj.1241)
Supplement: Table S4 — (Quantified using Pagel’s λ) (∗p < 0.05; ∗∗p < 0.01; and ∗∗∗p < 0.001). [file peerj-03-1241-s007.pdf]

**Trait**	**Pagel's λ**
Worker size	**0.97*** **
Worker polymorphism	**0.91*** **
Colony size	**0.97*** **
% Seeds in diet	**1.00*** **
% Insects in diet	**0.99*** **
% Liquid Foods in diet	**0.97*** **
Independent Colony Foundation	**0.92*** **
Polydomy	**0.78*** **
Polygyny	**0.85*** **
Strictly diurnal	**0.97*** **
Dominant	**0.99*** **
Foraging strategy	**1.00*** **
